# Responsible AI practice and AI education are central to AI implementation: a rapid review for all medical imaging professionals in Europe

**DOI:** 10.1259/bjro.20230033

**Published:** 2023-06-30

**Authors:** Gemma Walsh, Nikolaos Stogiannos, Riaan van de Venter, Clare Rainey, Winnie Tam, Sonyia McFadden, Jonathan P McNulty, Nejc Mekis, Sarah Lewis, Tracy O'Regan, Amrita Kumar, Merel Huisman, Sotirios Bisdas, Elmar Kotter, Daniel Pinto dos Santos, Cláudia Sá dos Reis, Peter van Ooijen, Adrian P Brady, Christina Malamateniou

**Affiliations:** 1 Division of Midwifery & Radiography, City University of London, London, United Kingdom; 2 Medical Imaging Department, Corfu General Hospital, Kontokali, Greece; 3 Department of Radiography, School of Clinical Care Sciences, Faculty of Health Sciences, Nelson Mandela University, Port Elizabeth, South Africa; 4 School of Health Sciences, Ulster University, Derry~Londonderry, Northern Ireland; 5 School of Health Sciences, Ulster University, Coleraine, United Kingdom; 6 University College Dublin, School of Medicine, Dublin, Ireland; 7 Medical Imaging and Radiotherapy Department, University of Ljubljana, Faculty of Health Sciences, Ljubljana, Slovenia; 8 Discipline of Medical Imaging Science, Sydney School of Health Sciences, Faculty of Medicine and Health, University of Sydney, Sydney, Australia; 9 The Society and College of Radiographers, London, United Kingdom; 10 Frimley Health NHS Foundation Trust, Frimley, United Kingdom; 11 Department of Radiology, University Medical Center Utrecht, Utrecht, Netherlands; 12 Department of Neuroradiology, University College London Hospitals NHS Trust, London, United Kingdom; 13 Department of Brain Repair and Rehabilitation, Institute of Neurology, UCL, London, United Kingdom; 14 Department of Radiology, University Medical Centre Freiburg, Freiburg, Germany; 15 European Society of Medical Imaging Informatics, Vienna, Austria; 16 European Society of Radiology, Am Gestade, Austria; 17 Department of Radiology, University Hospital Frankfurt, Frankfurt, Germany; 18 Department of Radiology, University Hospital Cologne, Cologne, Germany; 19 School of Health Sciences (HESAV), University of Applied Sciences and Arts Western Switzerland (HES-SO), Lausanne, Switzerland; 20 Department of Radiation Oncology/Data Science Center in Health (DASH), University of Groningen, University Medical Center Groningen, Groningen, Netherlands; 21 University College Cork, Cork, Ireland

## Abstract

Artificial intelligence (AI) has transitioned from the lab to the bedside, and it is increasingly being used in healthcare. Radiology and Radiography are on the frontline of AI implementation, because of the use of big data for medical imaging and diagnosis for different patient groups. Safe and effective AI implementation requires that responsible and ethical practices are upheld by all key stakeholders, that there is harmonious collaboration between different professional groups, and customised educational provisions for all involved. This paper outlines key principles of ethical and responsible AI, highlights recent educational initiatives for clinical practitioners and discusses the synergies between all medical imaging professionals as they prepare for the digital future in Europe. Responsible and ethical AI is vital to enhance a culture of safety and trust for healthcare professionals and patients alike. Educational and training provisions for medical imaging professionals on AI is central to the understanding of basic AI principles and applications and there are many offerings currently in Europe. Education can facilitate the transparency of AI tools, but more formalised, university-led training is needed to ensure the academic scrutiny, appropriate pedagogy, multidisciplinarity and customisation to the learners’ unique needs are being adhered to. As radiographers and radiologists work together and with other professionals to understand and harness the benefits of AI in medical imaging, it becomes clear that they are faced with the same challenges and that they have the same needs. The digital future belongs to multidisciplinary teams that work seamlessly together, learn together, manage risk collectively and collaborate for the benefit of the patients they serve.

## Introduction

### Rationale

Artificial intelligence (AI) implementation will bring AI from the lab to the clinic, to start benefiting the patients and easing workflows. To achieve this, different initiatives need to be put in place such as (a) responsible and ethical AI use, (b) relevant and customised training and education, and (c) multidisciplinary collaboration within the AI ecosystem. There is increasing work relating to the importance of involving patients to the design and implementation of AI tools but it goes beyond the scope of this paper and we hope to be able to develop this in a separate publication.

### Background

With the increasing rate of design and implementation of AI-enabled applications in healthcare, it is vital that the new technology is used responsibly and ethically^1^ to maximise benefits and mitigate risks. Radiology and radiography have attracted this technological revolution far more compared to other disciplines within healthcare.^2–5^ However, AI needs to be ethical and responsible, follow robust governance frameworks to guide safe and successful adoption, provide adequate education/training to key stakeholders, and all these are discussed below.

### Ethical AI

The concepts of responsible AI and AI ethics are deeply intertwined; AI cannot be considered responsible unless it conforms to certain AI ethical principles, underpinned by specific standards and regulations. Ethical concerns for AI in healthcare are complex, and often encompass the basic principles of: (i) beneficence and non-maleficence in relation to technical safety and clinical efficiency, (ii) data privacy and management and informed consent for data use, (iii) fairness/equity in their design and output, (iv) accountability in decision-making, (v) transparency and explainability.^3,6^

### Responsible AI

Responsible AI encompasses ethics but goes beyond the basic principles, extending into instilling integrity and trustworthiness into the conceptualisation, design, use, evaluation and monitoring of AI tools. These principles may relate to traceability, trustworthiness, establishing a robust ground truth for an AI tool, including a representative sample during training and testing of algorithms.^7–9^ These principles must underpin every stage of the AI lifecycle and be followed by all key stakeholders involved, from inception to implementation. The impact of AI within the medical imaging ecosystem is part of the ‘responsibility’ of AI designers, users, and consumers, as the downstream effects of AI may further influence new research or innovations in the area. Responsible and ethical AI are usually covered under one term called “AI governance”.

### Generic principles of responsible AI and AI ethics

AI governance models, non-specific to medical imaging, have been suggested to ensure AI is both responsible and ethical; governance is not a simple procedure and requires constant surveillance.^8,10^ It is essential to consider these principles at an individual level, but these must also be considered for wider populations and at a global socioeconomic level.^11^ Once the potential risks of AI are established, they can be protected against, and the undoubted benefits from AI can be maximised.^6^

### Unifying AI governance in medical imaging

Developing ethical and responsible AI is essential to establish and maintain clinician, patient and public trust and, therefore, facilitate implementation in clinical practice.^5,6,12^ Establishing robust governance for all stages of the AI lifecycle needs to involve all key stakeholders, from software developers, government bodies, clinical practitioners to patient interest groups, similar to what the Dutch AI coalition has achieved in the Netherlands.^8^ While most governance frameworks remain fragmented and contextualised, recent work in medical imaging and radiotherapy in the UK has proposed a unified governance framework, which underlines the importance of ethics, co-production with patients, and training on AI for all healthcare practitioners to facilitate its implementation.^13^ Similar work was conducted in other countries in the EU, such as the Innovation Funnel for Valuable AI in Healthcare by the Dutch Ministry of Health, Welfare and Sport^14^ which provides a tool identifying the legal and regulatory scope of action for eight steps in the innovation funnel.

### AI ethics guidelines for healthcare in Europe

In Europe, there are a variety of ethical guidelines and frameworks for the use of AI in healthcare,^15,16^ including data regulations, such as General Data Protection Regulation (GDPR).^17^ The European Commission published a set of non-binding ethical guidelines for trustworthy AI, non- specific to healthcare.^18^ Other institutions have also produced AI ethical guidelines,^19–24^ and academics have used these to compile local frameworks.^25–27^ A one-size-fits-all-approach to AI ethics in healthcare is proving too challenging, so there is, inevitably, a lot of fragmentation.^28^

The National Health Service (NHS) within the UK has established the ‘NHS AI Lab’; an organisation to aid acceleration of AI implementation.^29^ The NHS AI Lab comprises, among other important functions, an AI ethics initiative, which supports research that translates AI principles into practice, and helps build the evidence base required to mitigate risks and provide ethical assurance.^30^ This research could form the groundwork of robust European or global guidelines for AI ethics in healthcare. Furthermore, the British Standards Institute has drafted new AI standards specific to healthcare (“BS 30440 Validation framework for the use of AI within healthcare – Specification”), about to be published in Spring 2023, “applicable to products, models, systems or technologies whose function uses elements of AI, including machine learning (ML), and whose key function is to enable or provide treatment or diagnoses, or enable the management of health conditions, for the purposes of healthcare”.^31^

### AI ethics guidelines for medical imaging in Europe

Data used in medical imaging present the additional challenge of exhibiting a great variability,^32^ and lots of medical imaging data, usually in the universally accepted DICOM format, are unstructured.^33^ These additional challenges corroborate the need for rigorous guidance in medical imaging, compared to different specialties. Initially there were no standardised ethical guidelines for AI in medical imaging in Europe, with different countries, *e.g.* Italy and France, having produced country-specific AI ethical recommendations*.* ^34,35^ However, in 2019, this has changed with the joint, multisociety statement for AI ethics in medical imaging, including the European Society of Radiology (ESR) and the European Society of Medical Imaging Informatics (EuSoMII) as European partners.^3^ This joint statement offers unified ethical guidance, specific to the context of radiology. The key points of this statement are the need for the radiology community to recognise and mitigate the risks arising from AI and the need to acquire new skills to prepare for the digital future.^3^ While ethical considerations are required for patient and practitioner safety, caution is needed so they do not become hurdles to the development, creativity, and speed of implementation of AI technologies. These views are echoed in the joint statement by the European Federation of Radiographer Societies (EFRS) and the International Society of Radiographers and Radiological Technologists (ISRRT), as well as, the Society and College of Radiographers (SCoR) guidance, which highlight the need for radiographers to ensure that all AI applications are implemented in an ethical way.^36,37^

### The AI ecosystem in medical imaging

AI has the potential to change practice in medical imaging in different ways for different professionals. These changes in appointment scheduling, patient positioning, data acquisition, post-processing and segmentation or image analysis and interpretation may have tremendous impact on patient experiences and outcomes.^2,37,38^ It is definite that AI will take its position within the medical imaging ecosystem, where new roles, responsibilities and professional identities will develop, and the previous forms will go through a transitional phase.^39^ It is important for all medical imaging professionals (*e.g.* radiologists, radiographers, nuclear medicine practitioners, medical physicists) and other, increasingly needed scientists (*e.g.* data scientists/informaticians, biomedical engineers), to work together as a team.^3,36,40^ This unity must be manifested in both the theoretical principles and the practice and implementation of digital transformation.

### The importance of AI education

For a robust medical imaging ecosystem, both radiologists and radiographers need to be adequately educated in AI,^41^ including on AI ethics. For UK radiographers, AI is now included as a core competency for fitness to practice.^42^ Proportionate education might also be offered to patients, to make AI accessible, better understood, and trusted.

### Aim and objectives

The following work aims to discuss different AI implementation priorities,^12^ including: a) examine the different ethical frameworks about AI for radiologists and radiographers in Europe, and b) highlight the AI training provisions accessible to them. The synergies, challenges, and ways forward for the seamless collaboration between radiographers and radiologists will also be discussed.

## Methods

A rapid review^43^ was undertaken, with its design following an abbreviated, simplified version of review protocols, often purposedly omitting traditional methodological steps of a scoping or systematic review, to ensure timely data gathering from relevant literature.^44^ Rapid reviews can summarise extensive knowledge in a timely manner on fast developing topics, therefore suitable for AI-related work.^45^ Eligible papers were initially screened at abstract and title level and then included if they fulfilled the following eligibility criteria: a) related to AI (or synonyms, such as ML and deep learning) in radiology and radiography, b) pertaining to ethics, governance, and education/training, c) limited to Europe.

The search terms were pre-determined, as per Supplementary Table 1.

Supplementary Table 1.

A literature search for peer-reviewed literature was conducted within six databases (PubMed, MEDLINE, EBSCOhost, CINAHL, Taylor and Francis, and ERIC). Google Scholar was also used to ensure relevant grey literature was also included. Only literature written in English, published between 2012 and January 2023 was included in this search. Related literature on AI applications, ethics, education taking place outside Europe, or literature that did not specifically refer to radiography or radiology was excluded.

To understand the perspectives of the key European radiology/radiography learned societies, extra searches on their websites and related conferences were also conducted. Professional societies included were: the EuSoMII, the ESR, the SCoR, the EFRS, and the British Institute of Radiology (BIR).

After gathering all the eligible literature, a content analysis approach was applied, as a systematic, replicable, yet agile approach to understand, analyse, and interpret visual or verbal data.^46^ The compression of text, or phenomena progresses from defined generic concepts into common categories and, finally, overarching themes, in accordance with the texts’ characteristics.^47^ Content analysis is a focused process to categorise a large amount of text and ideas into a manageable number of groups.^48^ The interconnection between categories generates new concepts,^49^ can highlight different stakeholders’ perceptions, and provide an overview, suitable for a rapid review.^50^

## Results

**Figure 1.**
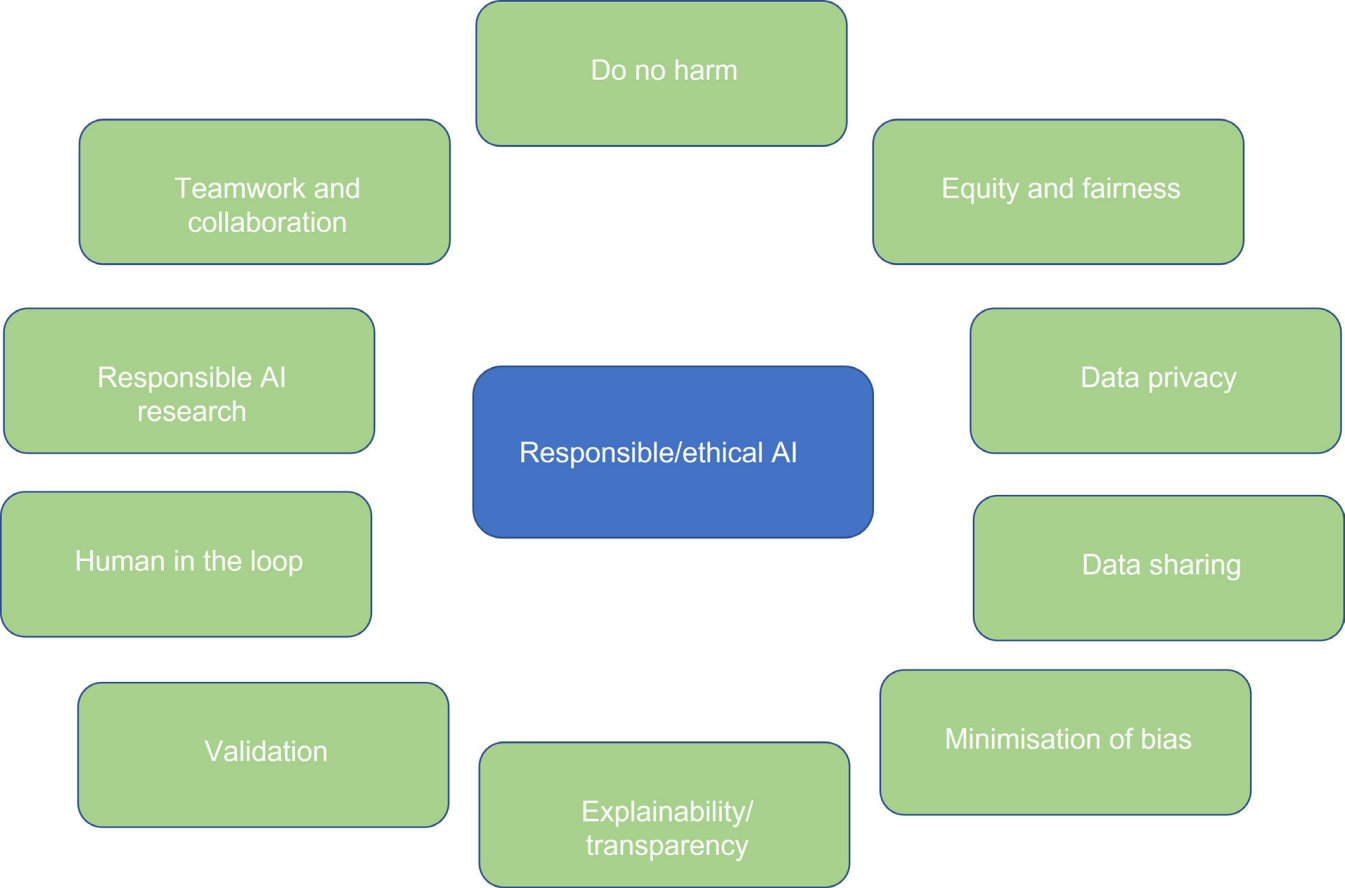
Responsible AI principles, based on the rapid review

In total, 19 papers in relation to AI ethics and medical imaging were sourced and more details of their key concepts can be found in Supplementary Table 2. This table summarises the currently available evidence that relates to ethical and responsible AI for radiology and radiography in Europe. These include the following principles (also presented in Figure 1), which are further commented on in discussion: do no harm, equity and fairness, data privacy, data sharing, minimisation of bias, explainability, external validation, human-in-the-loop approach, responsible AI research and AI teamwork.

Supplementary Table 2.

A rapid exploration of the available literature on AI education delivered in Europe, and of the websites of European universities and related AI industry, reflects a promising educational landscape for all relevant health professionals; bespoke information for patients is also available, albeit limited.

Supplementary Table 3 demonstrates the 24 different educational initiatives delivered in Europe for different healthcare professionals in medical imaging, summarising different information about delivery, cost, and target audience. Many of these opportunities can also be accessed by other professionals and organisations with a vested interest in AI. Finally, Supplementary Material 4 presents the related websites for access to these courses for those interested to learn more. These numbers are likely to change soon given developments in the field of AI but can be considered current up to June 2023.

Supplementary Table 3.

Supplementary Table 4.

## Discussion

The discussion below covers latest updates on AI education and governance, as well as the necessary synergies between radiographers and radiologists, and challenges and ways forward for AI implementation in medical imaging.

### AI governance

Our rapid review has identified that ethical and responsible AI is important for both radiologists and radiographers, as clinical practitioners. Radiographer-led papers appear somewhat later than the radiologist-led ones; this might relate to the early implementation of AI-enabled tools on image interpretation and diagnosis, that were available to radiologists, but, also, to the smaller critical mass of radiographer researchers.^51^ It might also convey their differing roles and liability in case of erroneous use and interpretation of AI technology. It is encouraging, though, to see that the same principles and concerns govern clinical practice of both professional groups. The principles outlined below apply to the clinical implementation of AI tools used by both radiologists and radiographers.

### Do no harm

Patient harm could result from the use of AI models, which have not been optimally trained and validated for the clinical setting at hand, either related to differences in the training population and clinical population or to the intended use. Practitioners might be liable for implementing these into clinical practice, in any context their role prescribes (whether in radiography or radiology).^3,52,53^ Responsible AI will help enhance patient care, societal, and practitioner acceptance, by promoting a culture of safety and trust.^5^

### Equity and fairness

Important differences in the use of AI have been found among high- and low-resource environments.^54^ AI developers and adopters must ensure that the benefits and the costs associated with the clinical use of AI will be equally distributed to all populations, thus enhancing the principle of fairness and justice.^55^

### Data privacy

Any data used for developing, training, and validating AI models need to be appropriately encrypted, secured and properly de-identified.^3,5,6,52,54^ Any sensitive information linked to data owners should be removed before it is shared and used, and appropriate processes should be established for data de-identification. Due to potential risks of data being re-identified, specific steps must be taken by organisations to eliminate these risks.^56^ The European GDPR should also be fully respected, to ensure legal and ethical use and sharing of data.^5,6^ In addition, organisations might take advantage of the block chain technology to improve cybersecurity.^56,57^ Differential privacy and homomorphic encryption are two more effective strategies to enhance security whilst also promoting innovation.^58^

### Data sharing

Centralisation of shared data using a single platform could result in a single point of data sharing policies and accountability.^56^ Rigorous data storage and data sharing policies will also help enhance patients’ privacy. To ensure data security, federated ML techniques can be used, where AI algorithms are distributed for local training at the place of data acquisition, instead of data being distributed to external companies.^58–60^

Informed consent is also a fundamental process linked to data sharing. Data owners (*i.e.* patients) have the right to decide on the use of their data, and all organisations should seek informed consent from patients before using their data for AI model development, training, or validation.^3,6,54,61^ This will also help to preserve patients’ autonomy, which is a fundamental principle of ethics.^18,52,62^ Finally, the same processes should be applied whenever patient data is required for AI research purposes, and this should be also stated during study reporting.^61^

### Minimisation of algorithmic bias

Algorithmic biases can occur when developing an AI model using a non-representative data set.^3,52,53^ These biases may include societal or financial factors, gender, sexual orientation, ethnicity, environment etc.^53^ These may further result in discrimination against certain underrepresented populations and also in health inequalities.^54^ When developing and training AI algorithms, it is essential to ensure that these algorithms use diverse data sets, ideally representing all populations. Diverse, large data sets are needed to minimise any data bias.^54,56^

### External, clinical validation

Before AI models have been clinically deployed, clinical validation is essential, ideally using new, unseen data, to ensure that the models’ performance is not compromised by differences raised by clinical environments.^5,6,37^ Clinical validation can be also performed by radiographers^39^ or radiologists,^56^ in their respective, distinct areas of practice.

### Explainability

AI algorithms should be explainable to end-users. When AI tools perform decision-making tasks, it is vital for end-users to be able to understand the rationale behind these decisions. Explainability is central to ethical and responsible AI, and it helps to build trust between practitioners, patients, and AI technology.^3,6,39,52,53^ Effort is required by vendors to develop transparent processes to support knowledge sharing with the wider scientific community.^35^

### Human-in-the-loop

Automation biases can occur when professionals overrely on the predictions made by AI models. This could potentially result in patient harm,^54^ thus being in contrast with the principles of responsible AI. Automation biases can be potentially exacerbated in low-resource environments, where there might be a lack of appropriately trained professionals to monitor the clinical effectiveness of AI models and identify any failures. A model that facilitates a ‘’human-in-the-loop’’ approach is optimal to minimise automation bias.^53^

### Responsible and ethical AI innovation and research

Further to ethical clinical practice, AI research and its dissemination must be always conducted in an ethical manner.^63^ AI researchers should ideally focus on prospective research studies.^61^ Also, diverse, multidisciplinary research teams working on AI projects can harness the different strengths and perspectives of the AI ecosystem.^39^ AI research studies should be transparent and allow open access to the code and training/validation data sets of the model.^61^ There is also a need to report data curation and data partitioning processes, data annotation strategies, and also to provide a detailed explanation of the model’s architecture and hyperparameters used.^61^ When reporting AI research studies, it is vital to ensure that all factors (human, algorithmic, environmental etc.) are clearly reported in a transparent way to minimise patient harm and research data waste.^64–66^ Finally, AI studies should be robust enough to allow reproducibility and generalisability of the results in all clinical settings, regardless of data sources.^61^

### AI teamwork and professional synergies

To ensure the ethical use of AI technologies in medical imaging, engagement of all key stakeholders is needed in all processes of the AI model’s lifecycle. Patient representatives are also essential,^5^ and they should be included as equal partners.^37^ This will also help to enhance the quality of the interaction between practitioners and patients.^62^ Another important step of making AI ethical and responsible is to involve multidisciplinary teams of key stakeholders in all AI procedures.^39,52^

### AI education and training

Our North American colleagues have recently worked to summarise the available AI educational provisions, outlining challenges and opportunities for Radiology.^67^ Europe has, in recent years, started to develop educational provisions for medical imaging professionals, to enable knowledge sharing, facilitate implementation and cultivate much-needed trust.^68^ More courses are being currently developed in Europe and ready to be launched in the academic year of 2023–2024, so this information will be changing very fast.

From Supplementary Table 3, it can be appreciated that the majority of education and training for medical imaging professionals and other healthcare scientists in Europe are of a continuing professional development nature, mostly delivered outside of formal academic education, sporadic in nature and not necessarily part of a lifelong learning journey.^69–85^ AI training overwhelmingly continues to be delivered through conference/congress programmes of learned societies^75^ and by commercial sponsors; the latter can cause concerns about how impartial or industry-agnostic the information can be. The overwhelming majority of the AI courses is primarily aimed at radiologists, with only few courses applicable currently to the radiographers’ remit.^86^ However, most course curricula have thematic overlaps for both professional groups and underline the common need for multidisciplinarity in content and delivery, to reflect the true AI ecosystems in medical imaging. The lack of alignment to the busy clinical schedules of radiologists and radiographers and their lack of time to engage with these courses, alongside associated costs, remain the major challenges of the delivery of AI-focussed educational provisions.^67,68,87^

AI-specific focussed conferences^70,71,73,74^ further underscore the efforts invested to enable the effective and efficient AI implementation, whilst ensuring patient safety, optimal care, and improved outcomes. The qualification-linked educational provisions are often linked to taught Master’s programmes^86,88,89^ or integrated at undergraduate level in a staggered manner.^87^ Those educational opportunities offered by commercial companies are frequently linked to their products and services^76,90^ which may leave knowledge gaps in the education and training of the practitioners that will work with different AI manufacturers in the clinical practice setting.

There are also a few platforms that primarily exist to share the latest information and insights about AI to those interested, whether professionals, organisations, researchers, or patients.^61,84,91^ One can thus argue that this contributes to continuous AI literacy development among professional communities and the general population in preparation for an AI-enabled world. There are multiple free education opportunities.^61,69,81,82,87,91^ However, other courses are fee-paying, which can limit accessibility to AI-related education and training.^70,71,73–75,77,83,85,86,88,89,92^

Evidence indicates that most AI education and training is offered in an online format, whilst some are in-person only. The duration of the current provisions ranges from 30 min to 1 year and others are self-paced. Most of the educational initiatives reviewed for this work are applicable to multidisciplinary audiences, followed by radiology-specific audiences. There is only one radiographer-specific module offered at present (Supplementary Table 3). It can be posited that this format can cultivate a stronger multidisciplinary workforce, where different parties have greater appreciations for others’ roles and therefore the patient care and management pathways and ultimately patient outcomes can be enhanced through cost-effective, timely, and appropriate actions taken by relevant practitioners.

There is a common thread regarding the content covered in the current AI education and training opportunities and programmes reviewed in this work. AI, ML and deep learning fundamentals, ethical considerations and societal impacts associated with AI, AI regulation, data sharing and preparation, explainable AI, workflow integration as well as specific clinical applications of AI relative to imaging modality or body area and structured reporting are common topics covered for both radiologists and radiographers. Radiomics, critical appraisal of AI literature, procurement, AI governance, prospective clinical trial development and implementation, scalability of AI innovations in healthcare settings and patient and practitioner acceptability are less frequently covered areas at present.

## Conclusions

The implementation of AI, as vital to harness the benefits of this new technology, relies on knowledge and trust. A comprehensive AI governance framework in medical imaging proposes this can be achieved through robust ethics, comprehensive, customised training and collaboration amongst healthcare practitioners, among other attributes. Responsible and ethical AI is vital to enhance a culture of safety and trust for healthcare professionals and patients alike. Educational and training provisions for medical imaging professionals on AI are central to the understanding of basic AI principles and applications. Education can facilitate the transparency of AI tools, but more formalised, university-led training is needed to ensure the academic scrutiny, appropriate pedagogy, multidisciplinarity and customisation to the learners’ unique needs are being adhered to. As radiographers and radiologists work together and with other professionals to understand and harness the benefits of AI in medical imaging, it becomes clear that they are faced with the same challenges and that they have the same needs. The digital future belongs to multidisciplinary teams that work seamlessly together, learn together, manage risk collectively and collaborate for the benefit of the patients they serve.
