# Supplementary material for: Responsible AI practice and AI education are central to AI implementation: a rapid review for all medical imaging professionals in Europe
Source: BJR Open. 2023 Jun 30;5(1):20230033. doi: 10.1259/bjro.20230033 (PMC10636340; doi:10.1259/bjro.20230033)
Supplement: Supplementary file 1 — Supplementary Table 1. [file bjro.20230033.suppl-01.docx]

**Supplementary table 1: describing search terms for different searches of the rapid review**

| Topic | Search terms |
| --- | --- |
| AI training and education | ((“AI” OR “artificial intelligence”) AND (“DL” OR “deep learning”)) AND (“ML” OR “machine learning”)) AND (“education” OR “training”) OR education OR programme OR courses)) |
| Ethical AI | ((Artificial intelligence OR ai OR A.I.) AND (radiographer(s) OR radiologic technologist OR practitioners OR radiology OR radiologists OR patients OR public OR data scientists OR data science OR health informaticians OR health informatics OR digital health OR medical imaging) |
