# Supplementary material for: Responsible AI practice and AI education are central to AI implementation: a rapid review for all medical imaging professionals in Europe
Source: BJR Open. 2023 Jun 30;5(1):20230033. doi: 10.1259/bjro.20230033 (PMC10636340; doi:10.1259/bjro.20230033)
Supplement: Supplementary file 2 — Supplementary Table 2. [file bjro.20230033.suppl-02.docx]

**Supplementary Table 2: Summary of European publications to support ethical and responsible AI**

| **Related literature** | **Concepts** |
| --- | --- |
| **Radiographers** | |
| The Society and College of Radiographers  policy statement: Artificial Intelligence.^51^ | “To promote safe and ethical practice that will enable better patient and service user outcomes.” |
| Artificial Intelligence and the Radiographer/Radiological Technologist Profession: A joint statement of the International Society of Radiographers and Radiological Technologists and the European Federation of Radiographer Societies.^36^ | “Ensure that all research involving AI systems is conducted  in an ethical way, communicating with patients on how their data may be used to develop and test AI.” |
| Artificial Intelligence: Guidance for clinical imaging and therapeutic radiography professionals, a summary by the Society of Radiographers AI working group.^37^ | Key recommendations for ethics as central to Ai adoption in   1. Clinical practice 2. Research 3. Education and training   -Engagement of radiographers into research  -Clinical validation from practitioners.  -Fairness  -Auditing from clinicians.  -Patients engaged as partners.  -Need for multidisciplinary teams that include key stakeholders from all areas. |
| Artificial intelligence in radiography: Where are we now and what does the future hold?^39^ | -Need for clinical validation from radiographers.  -Prospective multidisciplinary research studies.  -Accountability  -Explainability  -Transparency to gain patients’ trust. |
| **Radiologists** | |
| Artificial intelligence as a medical device in radiology: ethical and regulatory issues in Europe and the United States.^52^ | -Regulation  -Data protection  -Accountability |
| Artificial intelligence and medical imaging  2018: French Radiology Community white.^35^ | -Regulation (GDPR, French legislation).  -Data protection, pseudonymization.  -Research (France).  -Education (radiologists only).  -Algorithm biases.  -Ethics. |
| The ethical adoption of artificial intelligence in radiology.^53^ | -Centralization of shared data using a single platform.  -Consortiums to provide a single point of accountability and data sharing policies.  -Algorithmic bias, diverse data sets.  -Digitization of patient records and radiology reports.  -Data quality evaluation to make data AI-ready.  -Data owners’ informed consent.  -Data anonymization.  -Risks of data re-identification.  -block chain technology for cybersecurity.  -Benevolent AI.  -Radiologists to audit and supervise AI tools.  -Accountability  -Trust |
| From hype to hope to hard work: developing responsible AI for radiology.^5^ | -High-quality data must be used for reliable AI.  -A strong ethical framework and societal engagement is required.  -GDPR and privacy.  -Patient representatives are needed.  -New research methodologies should be used.  -Clinical validation is essential.  -Transparent and robust testing before deployment.  -Responsible AI will enhance patient care and societal acceptance. |
| Ethical considerations for artificial intelligence: an overview of the current radiology landscape.^54^ | -Algorithm biases  -Transparency  -Accountability  -Multidisciplinary stakeholders involved.  -Regulation (EU, USA, Canada).  -Data privacy  -Education  -Liability  -Explicability  -Autonomy |
| Responsibility beyond design: Physicians’ requirements for ethical medical AI.^55^ | -Accountability  -GDPR  -Entrustable Professional Activities  -Human interaction between radiologists and patients must be increased.  -Radiologists to communicate and explain the results to the patient.  -Explainable systems must make it possible to contest the decision of a system (contestability).  -Patients’ autonomy, beneficence, non-maleficence.  -Healthcare professionals to engage in specific training in order to avoid automation bias. |
| Integrating artificial intelligence into the clinical practice of radiology: challenges and recommendations.^56^ | -Algorithmic bias (may result in discrimination/inequalities)  -Automation bias  -Technical validation and clinical validation.  -Fairness  -Transparency  -Explainability  -Robustness, reproducibility, generalizability of algorithms.  -Need for diverse, large datasets, ideally from various institutions and geographical locations, to be shared.  -Data privacy  -De-identification  -Consent  -Accountability  -Differences in the use of AI in resource-rich and resource-poor environments.  -Need to ensure ethical governance and ethical auditing.  -Training needed to learn how to interact with and manage these technologies. |
| Artificial Intelligence in Radiology—Ethical Considerations.^6^ | -Data ownership and privacy.  -GDPR.  -Informed consent.  -Data anonymization.  -Data biases  -Transparency  -Interpretability  -Explainability  -Resource inequality  -Liability  -Explicability |
| FUTURE-AI: Guiding Principles and Consensus Recommendations for Trustworthy Artificial Intelligence in Medical Imaging.^57^ | -Fairness  -Universality  -Traceability  -Usability  -Robustness  -Explainability |
| A guide to good practice for digital and data-driven health technologies.^22^ | -Ethics.  -Understanding users’ needs.  -Ensure usability and accessibility.  -Validation testing.  -Ensure clinical safety.  -Data protection.  -Fairness, transparency.  -Cybersecurity.  -Algorithmic biases.  -Regulation (UK).  -Interoperability.  -Data standards. |
| DECIDE-AI: new reporting guidelines to bridge the development to implementation gap in clinical artificial intelligence.^58^ | -Clear and transparent reporting of human factors, technical requirements, safety profile of algorithms, clinical impact on its users’ decisions, to prevent harm and research waste. |
| Ethics of AI in Radiology: A Review of Ethical and Societal Implications.^59^ | -Data privacy. Data de-identified.  -Regulations for ethical use of sensitive information are needed.  -Transparency and explainability of models.  -Fairness and equality. Algorithms must be designed with the global community in mind. Validation should be performed with representative samples.  -Responsibility and accountability. End users should be taught how to use AI models and how to increase trust between them and AI.  -Justice. The benefits and costs of medical research should be equally distributed.  -AI should be trusted by clinicians and the patients.  -Bias resulting from data used to train and validate models (gender, sexual orientation, societal factors, financial, ethnic, environmental).  -Need to have annotated data to train the algorithms.  -Disparities will be exacerbated in radiology AI, since many institutions do not have the resources to manage complex AI systems, and they cannot afford them.  -Risk of automation bias will be increased in resource-limited settings, since they may not have a professional to monitor the model’s performance and identify its failure. A ‘’human-in-the-loop’’ approach is suggested.  -Specific challenges exist in clinically implemented AI models, as in some cases, no proof of improved clinical outcomes has been reported. Also, logistical challenges exist, quality control issues, and challenges with human barriers and algorithmic interpretability. |
| **Multi-professional bodies** | |
| Ethics of Artificial Intelligence in Radiology: Summary of the  Joint European and North American Multisociety Statement.^3^ | -Transparency  -Accountability  -Informed consent  -Data privacy  -Data ownership  -Algorithm biases  -Cybersecurity  -GDPR  -Trustworthiness  -Liability |
| Secure, privacy-preserving and federated machine learning in medical imaging.^60^ | Federated ML, differential privacy, and homomorphic encryption to ensure data privacy while also promoting technology. |
| MAIC–10 brief quality checklist for publications using artificial intelligence and medical images.^61^ | -Clinical need and added value to patients must be ensured to conduct AI studies.  -Need for observational studies with specific sample size calculations and level of confidence. Clearly defined inclusion/exclusion criteria.  -Ethically approved studies. Informed consent from data owners. Appropriate process for de-identification of data and for cybersecurity/privacy issues.  -Data curation (processes to extract, organise, clean, and pre-process data, software used with hyperparameters of transformations).  -Data annotation and ways to resolve discrepancies.  -Data partitioning (how it is split into training, tuning, validation sets).  -Need for an external validation data set.  -Detailed explanation of the AI model’s architecture, hyperparameters used, outputs to be predicted, and performance criteria used for the selected model.  -Robustness to allow consistent and reproducible results in clinical settings regardless of data sources.  -Transparency (open access to the code and data used to build and validate the model). Need to discuss funding role.  -Explainability (methods to make the model interpretable and understandable. Black box effects must be discussed. |
