# Supplementary material for: Responsible AI practice and AI education are central to AI implementation: a rapid review for all medical imaging professionals in Europe
Source: BJR Open. 2023 Jun 30;5(1):20230033. doi: 10.1259/bjro.20230033 (PMC10636340; doi:10.1259/bjro.20230033)
Supplement: Supplementary file 3 — Supplementary Table 3. [file bjro.20230033.suppl-03.docx]

**Supplementary table 3.** **An overview of some European AI education initiatives and opportunities for medical imaging professionals.**

| **Country of origin** | **Who offers the information/education/training** | **Education/ training programme/platform name** | **Year(s) offered** | **CPD/CME or qualification-linked or other professional development** | **Duration** | **Price** | **Target audience** | **Content covered** | **Mode of delivery** |
| --- | --- | --- | --- | --- | --- | --- | --- | --- | --- |
| Europe-wide | ESR/EusoMII | ESR AI masterclass | Start 2023, on demand | CPD linked to ESR | Self-paced | € 200 (excl. VAT)  € 150 (excl. VAT) for holders of an ESR Premium Education Package 2023 (initial offer, need to renew after a year) | Radiologists and trainees without need for prior Ai knowledge | This ESR Master Class gives you a fundamental education in AI, and includes 5 modules, from basics to clinical implementation, and will therefore advance your daily practice. |  |
| UK | City, University of London | Introduction to artificial intelligence for radiographers | Start date 2020, offered annually | Elective in master’s programmes and CPD standalone (30 UK credits, level 7) | 1 week | 1600GBP | This course is for recent radiography graduates, clinical practitioners, radiology managers, radiography researchers and educators who wish to further their understanding of the basic principles and applications of AI in Radiography and Medical Imaging | Basic AI concepts and terminologies; clinical applications of AI in projectional and cross-sectional imaging, reporting, ultrasound, mammography, interventional radiology and radiotherapy and nuclear medicine; Basic computer science fundamentals underpinning algorithms and associated workshop for hands-on work; Impact of AI on workflow in medical imaging; ethical considerations associated with AI; patient and healthcare acceptability of AI; industry-led workshops to introduce state-of-the-art AI applications and foster networking | 2020 online; 2021 onwards face-to-face |
| Germany | Technical University of Munich |  |  |  | 12 weeks (2 x 6 week blocks) | Free | Medical doctors, medical students, and non-MD researchers | Introduction to machine learning: historical context, systematic considerations, and basics of linear algebra; Introduction to artificial neural networks: what can AI learn? And basics of linear algebra; applying AI to imaging: special considerations for medical imaging; advanced learning methods with artificial neural networks: unsupervised learning; generative adversarial networks and medical image formats; critical appraisal of AI studies in radiology: reporting metrics and paper analysis; structured reporting in radiology; explainable AI in medical imaging; computational pathology; AI in dermatology; AI in neuroscience: ethical, legal and societal aspects; ethics in AI | Online only |
| Austria | EuSoMII | Webinars | Since 2018 & ongoing | Professional development | 30min to 1hour per webinar | Free | professional community with an interest in AI | Big Data, data analytics, machine learning applications to specific body systems/modalities, AI integration in workflow, data preparation for medical imaging, AI ethics, radiomics, explainability, procurement, structured reporting, trustworthy AI, monitoring and evaluation of AI in clinical practice | Online only |
| UK | BIR/ Health Education England | Webinars | Since 2023 | CPD/CME | Varying duration | Free | professional community with an interest in AI | Governance, AI basics, implementation, regulation, applications | Online only |
| UK | BIR/RCR | conference | 2023 | CPD/CME | 2 days | 100GBP to 495GBP | Radiologists (consultants and trainees), radiographers, physicists, oncologists as well as other healthcare professionals and those with an interest in AI | Topics will include: the use of AI data platforms, real world evidence of AI in clinical practice - head to toe, ecosystem of AI - regulations & procurement and future gazing | In-person only |
| UK | BIR/RCR | conference | 2022 | CPD/CME | 2 days | 75GBP to 475GBP | Radiologists (consultants and trainees), radiographers, physicists, oncologists as well as other healthcare professionals and those with an interest in AI | We will have presentations of success in real life working with radiology AI as well as the further steps and routes to adoption. Our contributors this year will share their experiences, tips and best practice in the implementation of AI. | In-person only |
| UK | BIR/RCR | AI in radiology: the main worries | 2019 | CPD/CME | 1 hour |  | Radiologists (consultants and trainees), radiographers, physicists, oncologists as well as other healthcare professionals and those with an interest in AI | briefly explore some of the issues that are worrying radiologists about AI. These include: Is the AI that is being developed really addressing clinically relevant issues? What sort of clinical problems would it be most useful for AI to tackle? There is a risk that financial and workforce pressures will drive the premature introduction of AI into clinical practice. How can radiologists be empowered to assess AI products that they may be asked to start using, to determine for themselves whether they are accurate and safe? What sort of regulation of AI software is currently in place, nationally and internationally, and is this sufficient? Do AI researchers have access to the vast amounts of good quality, standardised, curated, anonymised data that are needed to train, validate and test AI algorithms properly? | Online only |
| UK | BIR/RCR | Conference | 2019 | CPD/CME | 1 day | 65 GBP to 260GBP | Radiologists (consultants and trainees), radiographers, physicists, oncologists as well as other healthcare professionals and those with an interest in AI | provide an update on where we are with regards to artificial intelligence in the field of radiology. It will provide specific insights into the current and future applications of AI including deep learning in radiology, machine learning and computer-aided diagnosis with appropriate clinical applications. Educational aims: • To learn the fundamentals of imaging informatics and its clinical utility • To understand the latest technologies available for diagnosis using artificial intelligence techniques • To explore the implications of imaging informatics on the future of radiology | In-person only |
| UK | BIR/RCR | Conference | 2020 | CPD/CME | 2 days | 65GBP to 450GBP | Radiologists (consultants and trainees), radiographers, physicists, oncologists as well as other healthcare professionals and those with an interest in AI to join us at this exciting event. | Day one will cover talks on AI from a global perspective and the progressive work in this field being undertaken across continents. This will be followed by a session on AI in oncology in the afternoon, with eminent speakers in this field led by Royal Marsden Hospital.  Day two will cover clinical research-based talks being undertaken across the country to aid the healthcare industry with enhancing patient care / pathways and cutting-edge technology. | In-person only |
| Austria | ESR | Congress | 2023 | CPD/CME | 5 days | 100EUR to 1350EUR | radiologists (consultants and trainees), radiographers, physicists, oncologists as well as other healthcare professionals | Clinical applications, societal impact and ethics, workflow, data sharing and AI development, radiomics, use of AI in quality control testing, standards setting, | In-person |
| Indian company but UK-based training | Qure.ai | Qure.ai’s Artificial Intelligence Super User Certificate | started 2022 | CPD/CME |  |  | All medical professionals registered under The Health and Care Professionals Council are eligible for this training |  |  |
| Spain | ESR/ESOR | ESOR AI course 2019 | 2019 | CPD/CME | 2 days | 220EUR to 370EUR |  | The main focus of this AI event is the basic technical principles of AI and how they are applied to diagnostic imaging and clinical applications. The basic concepts of machine and deep learning will be explored, information about the type and consistency of imaging data processed by AI tools will be taught, and the main potential and emerging clinical applications will be revealed.  Two special sessions will bring a European flavour to the course, a panel discussion about the future of radiology and radiologists in the era of AI and one session open for SME companies to present themselves and showcase their product and the advantages of using their AI in different scenarios of their choice. | In-person only |
| Italy | ESR/ESOR | ESOR foundations course in AI in radiology 2020 | 2020 | CPD/CME | 2 days |  | Radiologists | The AI Foundation Course is aimed at providing the basis of AI and radiomics to the practicing radiologist and to illustrate what will be the professional impact, on ethics, workflow and education. A review of the developing clinical applications will help to understand how the clinical practice of radiologists will change in the near future. Internationally renowned experts will ensure a high quality teaching programme.   Learning Objectives to learn the basic principles of AI and Radiomics to review the developing clinical applications of AI and Radiomics to explore ethical aspects and new applications of AI in the modern radiological department | In-person only |
| Austria | ESR | ESR AI Blog | started in 2019 still ongoing | General update on AI developments | ongoing updates | free | Imaging professionals - full spectrum | This blog aims at bringing educational and critical perspectives on AI to readers. It should help imaging professionals to learn and keep up to date with the technologies being developed in this rapidly evolving field. | Online only |
| Spain | TMC Academy | Artificial intelligence in radiology workflow: from concept to experience (this is a fellowship) | 2022 | CPD/CME | 3 days |  | Radiologists | Refreshing on the basics of AI; introducing the range and diversity of the AI applications (Technography study); Showcasing some ways of implementing and working with AI; How to initiate working with AI: from the first day that it is coming to our desk, what do we need to do and how to work with it and what decisions are involved?; Experiencing how to work with different AI applications, on various clinical use-cases and under different working scenarios; AI tools related to different subspecialties (e.g., general radiology, skeletal, and prostate); AI tools with different functionalities and features (e.g., diagnosing, screening, segmenting, measurement); Different types of medical use cases (e.g., simple/complex); Under different working conditions (e.g., time-pressure, 1st vs. 2nd reader, individual vs. collective); Critical assessment of AI outcomes: issues, failures, suspicious cases  - Critical assessment of AI features/parameters/settings/configurations  - How to select your partner and shape it   - How to monitor your partner in the long-run (data shift);  - How to report and communicate the results of AI?  - Using AI in interactions with other medical colleagues and interdisciplinary discussions   - Interpretation and explanations to the patients; Organizational and workflow impacts and how to prepare them  - What infrastructure do you need?  - Whom do you need to involve?  - How to bring people on board?  - Ethical and Legal considerations (data sharing)?  - What training do you need to have? | Online only |
| Germany | Icon Institute | European Statistical training programme (ESTP) 2022 – AI for data science | 2022 and 2023 | CPD/CME | 5 days/3 days |  | data science | Demystification of AI and its relation to machine learning The cookbook recipe of creating an AI Real-world applications of state-of-the-art AIs Access to data, bias, ethics, regulatory frameworks Development of a no-code AI Demystification of AI and its relation to machine & deep learning The cookbook recipe of creating an AI Real-world applications of state-of-the-art AIs Access to data, bias, ethics, regulatory frameworks Development of no-code/low-code AI | In-person only |
| UK | University College London | MSc Artificial intelligence and Medical Imaging |  | Master's qualification 180 UK credits | 1 year | 16100 GBP | Suitable applicants will have a background in computer science, engineering or related field, looking to deepen their knowledge and specialise in applying it to healthcare | teaches the underlying concepts of Medical Imaging and Artificial Intelligence (AI), and how these technologies can be deployed in clinical environments, to help improve detection of disease and diagnosis from imaging data with the aim to enhance patient care.  **Compulsory modules**  Medical Imaging with Ionising Radiation  Biomedical Ultrasound  MRI and Biomedical Optics  Information Processing in Medical Imaging  Programming Foundations for Medical Image Analysis  MSc Research Project  Machine Learning in Medical Imaging  Applied AI in Medical Imaging  Applied Deep Learning  **Optional modules**  Computer-Assisted Surgery and Therapy  Medical Device Enterprise Scenario  Artificial Intelligence for Surgery and Intervention  Inverse Problems in Imaging  Computational Modelling for Biomedical Imaging  Computational MRI | In-person only |
| UK | City, University of London | MSc Artificial Intelligence |  | Master's qualification | 1 year | 10920 GBP | You are required to have a degree in computer science, computational neuroscience, mathematics, computer engineering, or natural sciences. | **Core modules**  Principles of Artificial Intelligence (15 credits) Programming and Mathematics for Artificial Intelligence (15 credits) Computational Cognitive Systems (15 credits) Agents and Multi-Agent Systems (15 credits) Deep Learning for Image Analysis (15 credits) Deep Learning for Sequence Analysis (15 credits) **Elective modules (choose 2)**  Deep Reinforcement Learning (15 credits) Explainable Artificial Intelligence: Ethical and Legal Challenges (15 credits) Industrial Artificial Intelligence (15 credits) **Individual Project (60 credits)** | In-person only |
| Belgium | EPF | European Patients Forum (EPF) AI knowledge hub |  | Information sharing/keeping updated with developments in AI |  | Free | Public, patients and health organisations and practitioners | source to learn more about Artificial Intelligence in Healthcare, with a particular focus on the patients’ perspective. The materials made available herein this section will help patient organisations and other interested stakeholders to better engage in debates, projects and initiatives on Artificial Intelligence in Healthcare, both at national and European level.   In the different sections of the resource point you will be able to find:   Information about the European Artificial Intelligence Fund;  EPF’s work on AI, including new initiatives, such as the Understanding AI Webinar Series, and previously published policy and capacity building resources;  External resources including key papers, reports, policy and legislative files, key events and capacity building activities. | Online only |
| Germany | Data Science Center in Health (DASH) at UMCG, in collab with Mediaire, Uniklinik Köln, University of Tartu. Københavns Universitet - University of Copenhagen, University of Groningen, 8D Games BV, FutureLearn, ArtiQ and OneVision Healthcare GmbH | How Artificial Intelligence Can Support Healthcare | Multiple start dates via FutureLearn still active | CPD/CME | 4 weeks | Free | doctors, nurses, GPs, and Biomedical researchers. It will also be of interest to patients, medical students, PhD students, and general AI enthusiasts. | Potential and limitations of AI in healthcare; Tackling the technical and regulatory challenges of AI; Ethical and social aspects of AI in healthcare; Practical examples and wrap-up; Explain the fundamentals of AI and its use in healthcare-based use-cases. Identify the requirements of implementation of AI in a clinical environment. Describe the impact of implementation of AI in a clinical environment. Evaluate the challenges and risks of AI in Healthcare. Discuss about AI and its use in healthcare in a practical and meaningful way. | Online only |
|  | GE Healthcare, KTH, LEITAT, EITH Health | HelloAI basic,advanced, professional and alumni. Four separate programmes | Started in 2018 and still ongoing | CPD/CME | 8 months | 49EUR to 399EUR | Available to everyone interested in AI in healthcare MSc students interested in healthcare PhD students, Researchers interested in healthcare Medical professionals, IT managers, Executives, Entrepreneurs (early career → Advanced, seniors → Professional programme) | Overview of AI in healthcare; operationalising AI in practice; AI innovation in healthcare organisations; data handling, preparing and distribution; scaling AI on a global platform. | Online only |
|  | AI4Europe project that has received funding from the European Union’s Horizon Europe research and innovation programme | European AI on demand platform |  | Keeping up to date with developments in AI |  |  | Any person with an interest in AI | The platform is a facilitator of knowledge transfer from research to multiple business domains.   The platform serves as a catalyst to aid AI-based innovation, resulting in new products, services, and solutions to benefit European industry, commerce, and society. By bringing people together, the community resource seeks to address the fragmentation of the European AI landscape and facilitate technology transfer from research to business. | Online only |
| Netherlands | Maastricht Univeristy | Artificial Intelligence 4 Imaging | 2022 ongoing | CME/CPD | 4 days | 850EUR to 3000 EUR | clinicians in medical imaging (e.g., radiologists, oncologists, neurologists, cardiologists, ophthalmologists, dermatologists, ENT surgeons) medical physicists with an interest in research medical imaging researchers computer scientists with an interest in medical imaging academics researching quantitative imaging | Radiomics, deep learning, explainable AI, auto segmentation and DL, synthetic data and neural networks, prospective clinical trials | In-person only |
| Netherlands | Quantib | webinars and variety of resources to consult |  | Other professional development |  |  | All professionals with an interest in AI | Applications of AI linked to Quantib's products | Online only |

*The grey shade blocks indicate that no specific information is available
