# Supplementary material for: Responsible AI practice and AI education are central to AI implementation: a rapid review for all medical imaging professionals in Europe
Source: BJR Open. 2023 Jun 30;5(1):20230033. doi: 10.1259/bjro.20230033 (PMC10636340; doi:10.1259/bjro.20230033)
Supplement: Supplementary file 4 — Supplementary Table 4. [file bjro.20230033.suppl-04.docx]

**Supplementary material 4: European courses on AI aimed at radiologists and radiographers (with weblinks)**

**(current as of June 2023)**

CPD at congresses/conferences/symposia

- EUSOMII <https://www.eusomii.org/>
- BIR <https://www.rcr.ac.uk/meeting/bir-ai-congress-2023>

<https://www.bir.org.uk/education-and-events/event-reviews/artificial-intelligence-where-we-are-now.aspx> <https://www.mybir.org.uk/CPBase__event_detail?id=a173Y00000GALT7QAP&site=a0N2000000COvFsEAL>

- ECR <https://www.myesr.org/congress/ai>
- UKIO <https://www.ukio.org.uk/programme-2022/>

Radiographer-specific courses

- City University of London <https://www.city.ac.uk/prospective-students/courses/professional-development/introduction-to-artificial-intelligence-for-radiographers>
- Qure.ai super user training certificate endorsed by CoR, also open to other HCPC registered professionals <https://qure.ai/gain-cor-endorsed-super-user-training/>
- Society and College of radiographers AI webinars and AI podcasts (available from September 2023) <https://www.collegeofradiographers.ac.uk/education>

Radiology-specific courses and webinars

- ESR Masterclass in AI 2023 <https://www.myesr.org/masterclass-in-ai>
- BIR education essentials 2023 <https://bir.org.uk/education-and-events/ai-essentials-webinar-series.aspx>
- Society and College of radiographers AI webinars and AI podcasts (available from September 2023) <https://www.collegeofradiographers.ac.uk/education>
- ESOR foundations course in AI in radiology 2020 <https://healthmanagement.org/c/imaging/event/esor-foundation-course-on-artificial-intelligence-in-radiology>
- ESOR AI course 2019 <http://www.myesr.link/Mailings/ESORAInm/?utm_source=ESOR+non+member&utm_campaign=0934231224-EMAIL_CAMPAIGN_2019_02_04_08_44_COPY_03&utm_medium=email&utm_term=0_1560fe400b-0934231224-83993449>
- ESR AI Blog <https://ai.myesr.org/education/>
- Artificial intelligence in radiology workflow: from concept to experience <https://academy.telemedicineclinic.com/fellowships/3391/artificial-intelligence-in-radiology-workflow-from-concept-to-experience-nov-2022/>

Data science specific course

- European Statistical training programme (ESTP) 2022 – AI for data science <https://ec.europa.eu/eurostat/cros/content/artificial-intelligence-data-science-2022_en>
- The heart of AI: AI and data science training hub in the center of Europe, Kozminski Uni <https://www.kozminski.edu.pl/en/heart-ai-ai-and-data-science-training-hub-center-europe-hai>
- Artificial Intelligence and Medical Imaging MSc

<https://www.ucl.ac.uk/prospective-students/graduate/taught-degrees/artificial-intelligence-and-medical-imaging-msc>

Information for patients

- European Patients Forum (EPF) AI knowledge hub <https://www.eu-patient.eu/ai-knowledgehub/>

Multidisciplinary courses/information for professionals and managers

- <https://eithealth.eu/programmes/aiprohealth/>
- HelloAI <https://www.helloaiprofessional.com/>
- HelloAI RIS <https://helloaionline.com/>
- European AI on demand platform <https://www.ai4europe.eu/>
- Artificial Intelligence 4 Imaging <https://www.ai4imaging.org/>
